# The impact of work–family conflict on early childhood teachers’ occupational well-being: the chain mediating role of psychological empowerment and job crafting

**DOI:** 10.3389/fpubh.2024.1513514

**Published:** 2025-01-08

**Authors:** Liqun Wang, Tianqi Qiao, Xinxin Wang, Chen Wang, Pingzhi Ye

**Affiliations:** College of Education, Guangzhou University, Guangzhou, Guangdong, China

**Keywords:** work–family conflict, occupational well-being, psychological empowerment, job crafting, early childhood teachers

## Abstract

**Purpose:**

The occupational well-being of early childhood teachers, as a crucial measure of the stability of the early childhood workforce, is increasingly becoming a core topic of interest within the education system. Work-related stressors, particularly work–family conflict, have drawn significant attention for their impact on the occupational well-being of early childhood teachers, becoming a prominent issue in the education field. However, current research rarely explores the relationship between these factors and the underlying mechanisms involved. Therefore, this study aims to investigate the relationship between work–family conflict and the occupational well-being of early childhood teachers and the mediating role of psychological empowerment and job crafting.

**Methods:**

This study conducted a survey involving 1,200 early childhood teachers from Guangdong Province, China, using personal information forms and four scales. The collected data were processed and analyzed using SPSS 27.0.

**Results:**

Work–family conflict showed a significant negative correlation with early childhood teacher’s occupational well-being (*β* = −0.268, *p* < 0.001). Psychological empowerment (indirect effect size = −0.049) and job crafting (indirect effect size = −0.019) partially mediated the relationship between work–family conflict and occupational well-being. Furthermore, psychological empowerment and job crafting played a chain mediating role between work–family conflict and occupational well-being (indirect effect size = −0.036).

**Conclusion:**

This study reveals the underlying mechanisms by which work–family conflict affects early childhood teachers’ occupational well-being. The findings demonstrate that work–family conflict has a direct and negative impact on the occupational well-being of early childhood teachers. Psychological empowerment and job crafting both partially mediate the relationship between work–family conflict and occupational well-being among early childhood teachers, and they also function in a serial mediating role within this association. The study provides crucial evidence supporting the significant impact of work-related stressors on early childhood teachers’ occupational well-being, serving as a reference for policymakers and educators in developing interventions targeting occupational well-being.

## 1 Introduction

In recent years, the continued advancement of early childhood teachers’ occupational well-being has gradually emerged as a focal issue in global education research (1–3). In 2018, the Central Committee of the Communist Party of China and the State Council issued the “Opinions on Comprehensively Deepening the Reform of Teacher Development in the New Era,” stating, “By 2035, the entire society will respect teachers and education, allowing educators to feel happiness in their positions, fulfillment in their careers, and honor within society, making teaching an admired profession” (4). In January 2020, the Organization for Economic Co-operation and Development (OECD) released “Teachers’ Well-being: A Framework for Data Collection and Analysis,” which, for the first time, included occupational well-being of teachers as a significant component in the 2021 Program for International Student Assessment (PISA) (2). High levels of occupational well-being are crucial not only for the stability of the education system, resulting in better work performance, enhanced innovative behaviors, and lower turnover intentions but also directly impact children’s growth and development. It is the most important internal factor in promoting students’ success, satisfaction, and achievement (5–9). Conversely, low levels of occupational well-being among teachers can lead to several negative consequences, such as high turnover intentions, teacher shortages, and diminished attractiveness of the profession (2). Specific research indicates that in the United States, around 30% of teachers leave within 5 years of graduation. In France, Spain, and Sweden, less than 10% of teachers perceive teaching as valuable, and the profession’s appeal is gradually declining (10, 11). In this turbulent educational environment, the organizational efficiency of the education system is significantly impacted, and the quality of children’s education is challenging to ensure (12, 13). The current state of early childhood teachers’ occupational well-being is even more disheartening. A series of studies have consistently shown that the mental health and occupational well-being of early childhood teachers are often faced with significant challenges. Many early childhood teachers are in poor mental health, even accompanied by mild, moderate, or severe mental problems (14–19).

A substantial body of research has confirmed the significant impact of work-related factors on the occupational well-being of early childhood teachers (20, 21). Among these factors, work stressors, particularly work–family conflict, have garnered widespread attention (22–24). Work–family conflict primarily arises from the interplay and mutual influence between work and family domains. Specifically, when early childhood teachers gain resources in the work (or family) domain that enhance their role performance in the family (or work) domain, a mutual enrichment between work and family occurs. For example, the empathy and emotional insight cultivated by early childhood teachers in their work enable them to more keenly perceive the emotional needs of their family members in family life, thereby providing timely emotional support and comfort, and fostering harmony and stability within the family. Concurrently, understanding, encouragement, and shared responsibilities from family members can effectively alleviate the work-related stress of teachers (24). However, when role pressures and competition for resources coexist with inconsistencies between personal goals and family expectations, coupled with a lack of communication and understanding, work–family conflict may be triggered. On one hand, the job demands and pressures faced by early childhood teachers may impinge upon their family life. Specifically, early childhood teachers are required to meet diverse job demands (25). They are not only responsible for nurturing and educating young children but also face extended working hours and more frequent supervisory inspections. Along with the ever-increasing educational demands, early childhood teachers may still have to deal with various work-related tasks after returning home (26, 27), such as communicating with parents and paperwork. These job requirements make it difficult for early childhood teachers to achieve a balance between work and family life, thereby reducing the time and energy available for family companionship and fulfilling family responsibilities, which may lead to dissatisfaction and complaints from family members. Driven by stress, early childhood teachers often experience fatigue and negative emotions. These pressures and negative emotions can spill over into their family domain, potentially leading to conflicts among family members (28, 29). On the other hand, family issues can also negatively affect the work status of early childhood teachers. For instance, when family members fall ill or require care, early childhood teachers may become distracted, preventing them from fully engaging in their work, which can subsequently impact their work efficiency and the quality of education provided (30). Over time, the stress resulting from work–family conflict interacts within both domains, making it increasingly difficult for teachers to balance work demands with family life. The significant stress that arises from this conflict is likely to induce job burnout and health issues among early childhood teachers, thereby seriously jeopardizing their occupational well-being (31–34). Moreover, such impacts may be direct or indirect, mediated through the influence on other factors.

Additionally, influenced by traditional Confucian ideology, Chinese society believes women should prioritize family over career more than men, leading to a higher likelihood of work–family conflict for Chinese women (35). Compared to primary and secondary school teachers, women make up a vast majority of early childhood teachers (36). They play the roles of teachers, mothers, and daughters, experiencing more work–family conflict. With increasing conflict intensity, early childhood teachers increasingly suffer from emotional fatigue, work-related stress, and health problems, jeopardizing their occupational well-being (37–42).

In summary, early childhood teachers’ work–family conflict and occupational well-being urgently require urgent attention (3). However, the current academic field still lacks exploration of the relationship and mechanisms between work–family conflict and occupational well-being. Therefore, based on previous research and utilizing the Job Demands-Resources model (JD-R) as the main analytical framework, this study positions work–family conflict as a critical factor. It aims to explore the impact of work–family conflict on the occupational well-being of early childhood teachers and its underlying mechanisms, providing valuable insights and practical recommendations for enhancing early childhood teachers’ occupational well-being and promoting their physical and mental health.

### 1.1 Work-family conflict and occupational well-being

Work–family conflict is a type of role conflict that occurs when an individual’s work role prevents effective functioning in their family role, or vice versa (43).

The Job Demands-Resources model effectively explains the impact of work–family conflict on the occupational well-being of early childhood teachers. Specifically, when individuals face intense work pressure, it can permeate into their family domain, leading to work–family conflict. This conflict subsequently triggers stress, fatigue, and burnout, affecting both physical and mental health, as well as job and life satisfaction, thereby influencing occupational well-being and related factors. This process has been corroborated in research (31–34). Subsequent studies involving primary, secondary, and university teachers have reinforced this perspective, finding a significant negative correlation between work–family conflict and occupational well-being (23, 24). Research on early childhood teachers similarly found that work–family conflict positively affects their turnover intention, which is closely associated with occupational well-being (2, 44). In summary, existing studies have confirmed from various perspectives the negative impact of work–family conflict on occupational well-being. However, research specifically examining the relationship between work–family conflict and early childhood teachers’ occupational well-being and the mechanisms involved remains limited, warranting further exploration.

### 1.2 The mediating role of psychological empowerment

Psychological empowerment is defined as a motivational structure manifested in four cognitions: meaning, competence, self-determination, and impact, reflecting a proactive orientation toward one’s work role (45). For early childhood teachers, psychological empowerment holds significant importance, as a high level of psychological empowerment may imply that work is meaningful, confidence in one’s educational capabilities, autonomy in the workplace, and belief in one’s ability to positively impact the organizational environment (45).

When confronted with work–family conflict, psychological empowerment emerges as a personal resource that safeguards early childhood teachers from the adverse effects of work demands and their associated impacts by nurturing positive work attitudes, such as increasing job engagement (45). However, the Job Demands-Resources (JD-R) model posits that high job demands and limited job resources lead to energy depletion and may weaken employees’ motivation (46, 47). Relevant research supports the aforementioned viewpoint (48). It is noteworthy that the group of early childhood teachers frequently confronts arduous workloads, yet they continue to grapple with issues such as low salary treatment, lack of status, and insufficient public recognition (37, 49). These circumstances may contribute to a lower level of psychological empowerment among early childhood teachers. When confronted with work–family conflict, the subsequent stress may further diminish their psychological empowerment. Furthermore, psychological empowerment may positively influence the occupational well-being of early childhood teachers (50, 51). As a psychological motivation construct, psychological empowerment acts as a personal resource, boosting positive job outcomes, such as job engagement and satisfaction, thus enhancing occupational well-being of early childhood teachers (46, 47). Subsequent research has also confirmed the aforementioned viewpoint, indicating that psychological empowerment can positively predict the occupational well-being of early childhood teachers (52). Therefore, psychological empowerment may mediate the impact of work–family conflict on occupational well-being of early childhood teachers.

### 1.3 Mediating effect of job crafting

Job crafting refers to changes employees make to align job demands and resources with their personal skills and needs (53). For early childhood teachers, job crafting can be viewed as a proactive coping strategy in response to changes in the work environment (54).

Work–family conflict may negatively impact job crafting. The JD-R model asserts that job crafting is only possible when job demands are manageable (55). Specifically, work–family conflict can be viewed as a job demand (24, 56). When work demands are manageable and early childhood teachers possess adequate work and personal resources, job crafting enables them to adjust their work tasks and relationships according to their interests and abilities. Through job crafting, early childhood teachers not only enhance their work efficiency and increase their job satisfaction but also mitigate the encroachment of family time or emotional exhaustion caused by excessive work stress, thereby contributing to the harmony and stability of their family life (57). However, when the demand for work is so high that the workload does not allow early childhood teachers to find the necessary energy to meet the demand or when individuals are likely to be concerned about losing the resources they already have, they will not engage in job crafting behavior (58–61). This viewpoint has also been substantiated by relevant studies (62). Secondly, one of the defining characteristics of the profession of early childhood teachers is the challenge of facing multiple role demands (63). Influenced by China’s unique socio-cultural background, when confronted with work–family conflict, early childhood teachers may adopt attitudes and responses toward job crafting that are entirely distinct from those of other professions, to the extent that they may not even consider engaging in it. However, research on the impact of work–family conflict on job crafting among early childhood teachers remains scarce, warranting further investigation. Furthermore, job crafting is likely to positively predict the occupational well-being of early childhood teachers. Research on early childhood teachers found that job crafting enables alignment of work with teachers’ abilities, needs, and preferences, which in turn contributes to enhancing their work experiences, mitigating occupational dysfunction, and ultimately increasing their occupational well-being (64). Another study has also demonstrated that job crafting can enhance early childhood teachers’ sense of self-efficacy, work control, and job engagement, and teachers with these characteristics often possess higher levels of occupational well-being (3, 64). Thus, job crafting may be another mediator in the relationship between work–family conflict and occupational well-being of early childhood teachers.

### 1.4 The chain mediating role of psychological empowerment and job crafting

To deepen our understanding of the impact of psychological empowerment on job crafting among early childhood teachers, we can attempt to describe their combined effects from the perspective of internal and external resources. Psychological empowerment can endow individuals with a positive mindset and motivation, which we can consider as an internal resource (45). Job crafting, on the other hand, can be viewed as an external resource or strategy, as it involves adjustments to the work environment and tasks to accommodate individual needs and capabilities (55). Abundant internal resources may help to motivate early childhood teachers to actively seek and accumulate external resources. According to Self-Determination Theory (SDT), when individuals are psychologically empowered, they exhibit a more positive sense of work meaning, work competence, autonomy, and impact, which in turn makes them more likely to engage in proactive work behaviors such as job crafting (53, 65–67). When early childhood teachers are psychologically empowered, they may display a more positive work status and are more likely to optimize their work environment and tasks through job crafting to better adapt to changes in the work environment and tasks. Existing research has also demonstrated the positive effects of psychological empowerment on job crafting (68, 69).

Integrating theory and existing research, we can infer the potential chain mediating role of psychological empowerment and job crafting between work–family conflict and the occupational well-being of early childhood teachers. According to the JD-R model, high job demands and limited resources lead to energy depletion and decreased motivation. Resource depletion and reduced motivation lower psychological empowerment, making early childhood teachers less inclined to engage in proactive behaviors like job crafting to cope with high job demands and the resulting stress and negative outcomes, reducing occupational well-being (45, 57, 58, 70). In summary, psychological empowerment and job crafting may form a chain mediating mechanism through which work–family conflict affects occupational well-being.

### 1.5 The current study

Work–family conflict significantly impacts early childhood teachers’ occupational well-being, and its mechanisms are likely multifaceted and complex. Although prior studies have provided theoretical explanations and empirical research on work–family conflict, given the distinct nature of early childhood teachers and the complexity of how work–family conflict influences their well-being, it is essential to explore the specific pathways of this effect. Additionally, considering the increasing stress faced by early childhood teachers and the growing concern over their well-being (1), finding effective strategies to enhance early childhood teachers’ well-being has become an urgent task in the field of early childhood education. As work-related stressors are key negative factors affecting their well-being, they should be the focal point of researchers (71, 72). It is noteworthy that theory and related research suggest that psychological empowerment and job crafting are pivotal variables in this influence mechanism. Specifically, work–family conflict may initiate a health-impairment process that adversely affects early childhood teachers’ occupational well-being by negatively influencing their psychological empowerment or job crafting, respectively. This process can also lead to early childhood teachers’ reluctance to engage in job crafting by diminishing their level of psychological empowerment, ultimately having an adverse impact on their occupational well-being (57, 58, 73).

**Figure 1:**
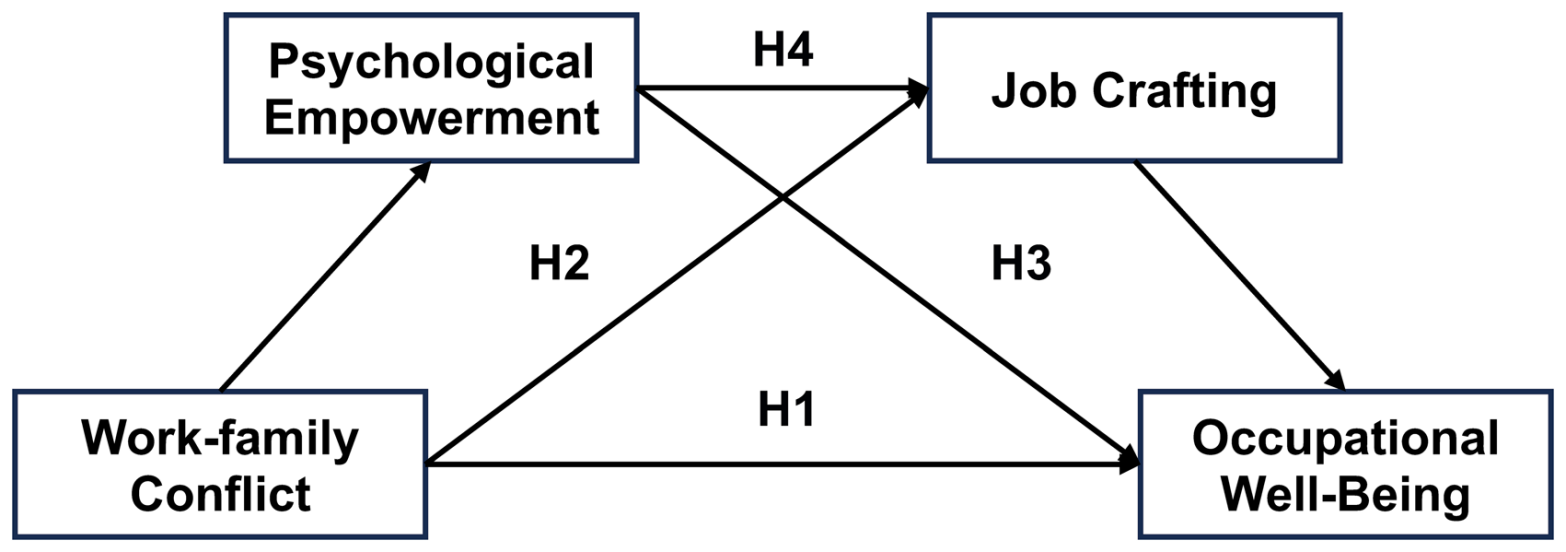
Hypothesized conceptual model.

Therefore, this study thoroughly explores the impact mechanism of work–family conflict on the occupational well-being of early childhood teachers, examining the potential mediating roles of psychological empowerment and job crafting. This research will further contribute to advancing related fields and offer new evidence, providing practical insights into improving early childhood teachers’ occupational well-being. Based on existing studies, we constructed a hypothesized model (Figure 1) and proposed the following hypotheses:

*H1*: Work–family conflict is negatively correlated with early childhood teachers’ occupational well-being.

*H2*: Psychological empowerment mediates the impact of work–family conflict on early childhood teachers’ occupational well-being.

*H3*: Job crafting mediates the impact of work–family conflict on early childhood teachers’ occupational well-being.

*H4*: Psychological empowerment and job crafting jointly form a chain mediating mechanism between work–family conflict and occupational well-being.

## 2 Methods

### 2.1 Participants and procedure

This cross-sectional study collected data through convenience sampling. In March 2024, we distributed an online survey to early childhood teachers in Guangdong Province, China. The recruitment period for the questionnaire spanned from March 14th to March 21st, 2024. Initially, we distributed 1,456 questionnaires and recovered 1,443. Inclusion criteria included (1) early childhood teachers in Guangdong Province, China, and (2) teachers willing to participate and accurately complete the survey. Exclusion criteria were (1) missing data, (2) more than 85% of answers being the same and (3) survey completion time < 4 min. After data filtering, 243 questionnaires were deemed invalid, leaving 1,200 valid questionnaires, yielding a response rate of 83.2%. The average age of participants was 32.95 (SD = 7.85, ranging from 17.00 to 54.00). The sample comprised 34 males (2.8%) and 1,166 females (97.2%), with an average daily working time of 9.13 h (SD = 0.89, ranging from 7.50 to 10.50).

This research was conducted in accordance with the ethical principles of voluntary participation and the Helsinki Declaration, obtaining informed consent. Prior to completing the questionnaire, we informed the early childhood teachers that the data collected would be anonymized and kept strictly confidential, and we inquired whether they were willing to participate in the survey. If the early childhood teachers selected “I consent to participate in this survey,” it indicated agreement, and the questionnaire page was subsequently displayed. If the early childhood teachers selected “I do not wish to participate in this survey,” the survey was terminated. Participants were allowed to withdraw at any time. This study has received approval from the Ethics Committee of Guangzhou University.

### 2.2 Measures

#### 2.2.1 Personal information form

The form includes basic information like teachers’ gender, age, and daily working hours.

#### 2.2.2 Work–family conflict

The Chinese adaptation of the Work–Family Conflict Scale was used (74). Initially developed by Carlson et al. in 2000, it contains 18 items across six dimensions of conflict, assessing three forms of work–family conflict (time, strain, and behavior) and two directions (work interfering with family and family interfering with work) (75). All items use a five-point Likert scale ranging from 1 (strongly disagree) to 5 (strongly agree). The scale has shown good reliability and validity in the Chinese population (76). In this study, Cronbach’s alpha coefficient was 0.95.

#### 2.2.3 Kindergarten teachers’ occupational well-being scale

We used the revised version by Wang (77). The scale is based on Xiuzhi Liu’s “Kindergarten Teacher Workplace Well-being Questionnaire” and incorporates the characteristics of mainland China’s economic development and early childhood teachers. It consists of four subscales (psychological well-being, emotional well-being, social well-being, and cognitive well-being) with 15 items. Each item uses a five-point Likert scale ranging from 1 (strongly disagree) to 5 (strongly agree), with higher scores indicating higher well-being. Cronbach’s alpha coefficient was 0.93.

#### 2.2.4 Psychological empowerment scale

The Chinese adaptation of the Psychological Empowerment Scale (PES) was used (78). Initially developed by Spreitzer (45), it includes four dimensions (meaning, competence, self-determination, and impact) with 12 items. The survey uses a five-point Likert scale, from 1 (strongly disagree) to 5 (strongly agree). The scale has demonstrated good reliability and validity in the Chinese population (79). In this study, Cronbach’s alpha coefficient was 0.95.

#### 2.2.5 Job crafting scale

The Chinese adaptation of the Job Crafting Scale includes 20 items (80). Initially developed by Tims et al. (81), it consists of four dimensions: increasing social job resources, increasing structural job resources, increasing challenging job demands, and decreasing hindering job demands. The scale uses a five-point Likert scale, from 1 (strongly disagree) to 5 (strongly agree). It has shown good reliability and validity in the Chinese population (82). In this study, Cronbach’s alpha coefficient was 0.92.

### 2.3 Data analysis

The study collected and analyzed data using SPSS 27.0 software. First, descriptive statistics (mean and standard deviation) and standardization were performed on the independent variable (work–family conflict), mediating variables (psychological empowerment, job crafting), and the dependent variable (occupational well-being). Second, Pearson correlation analysis was used for bivariate analysis to explore correlations between work–family conflict, mediating variables, and problem behaviors. Correlations between variables were expressed using the correlation coefficient r, and *p* < 0.05 indicates that the correlation is statistically significant. Next, Model 6 in the SPSS macro program PROCESS was used to test the mediating roles of psychological empowerment and job crafting in the relationship between work–family conflict and occupational well-being (83). Standardized path coefficients were represented by *β*, with *p* < 0.05 indicating statistical significance. PROCESS is an observed variable OLS (Ordinary Least Squares) and logistic regression path analysis modeling tool (83). It implements moderation or mediation analysis, as well as their combination in an integrated conditional process model (i.e., mediated moderation and moderated mediation) (84). Model 6 within the PROCESS macro is specifically designed for testing sequential mediation effects, which aligns with our research hypotheses. In this model, there exists an orderly sequence among the mediator variables, where the effect of one mediator is transmitted to the next, ultimately influencing the outcome variable. Prior to applying this model, we generated histograms for the studied variables using SPSS, and the results demonstrated that the data followed a normal distribution. Additionally, we verified the linear relationships among the variables in the correlation analysis section. Finally, previous studies have shown that the bias-corrected percentile Bootstrap method is more effective than the traditional Sobel method (85). Therefore, we used the bias-corrected bootstrap method on 5,000 samples to test the mediation hypotheses while including gender, age, and daily work hours as covariates. An indirect effect was considered statistically significant if the 95% confidence interval (CI) did not include 0.

## 3 Results

### 3.1 Common method biases tests

All data were collected through self-reported questionnaires, which could result in common method biases that may affect the results. To minimize this potential bias, measures such as ensuring anonymity, separate arrangements for different questionnaires, and emphasizing data confidentiality were taken. Moreover, Harman’s single-factor test was used for post-hoc statistical testing (86). The results showed that 11 factors had eigenvalues greater than 1, and the largest factor explained 30.98% of the variance (<40%), indicating that the data were not significantly affected by common method biases.

### 3.2 Preliminary analysis

Pearson correlation analysis was conducted to investigate the relationships between key demographic variables (gender, age, and daily work hours) and work–family conflict, as shown in Table 1. Work–family conflict was negatively correlated with psychological empowerment, job crafting, and occupational well-being (*p* < 0.001); psychological empowerment was positively correlated with job crafting and occupational well-being (*p* < 0.001); and job crafting was positively correlated with occupational well-being (*p* < 0.001). Additionally, gender was positively correlated with job crafting (*p* < 0.05). Age was significantly correlated with daily work hours (*p* < 0.05), work–family conflict (*p* < 0.01), psychological empowerment (*p* < 0.01), job crafting (*p* < 0.05), and occupational well-being (*p* < 0.001). Daily work hours were significantly correlated with work–family conflict (*p* < 0.001), psychological empowerment (*p* < 0.05), job crafting (*p* < 0.05), and occupational well-being (*p* < 0.001).

**Table 1:** Descriptive statistics and correlation analysis.

	Mean	SD	1	2	3	4	5	6	7
1. Gender	–	–	–						
2. Age	32.95	7.85	−0.03	–					
3. Daily working hours	9.13	0.89	0.01	−0.07*	–				
4. Work–family conflict	2.85	0.72	0.04	−0.09**	0.19***	–			
5. Psychological empowerment	3.84	0.55	0.06	0.09**	−0.06*	−0.14***	–		
6. Job crafting	3.96	0.45	0.07*	0.07*	−0.06*	−0.15***	0.71***	–	
7. Occupational well-being	3.84	0.55	0.04	0.18***	−0.15***	−0.39***	0.67***	0.68***	–

*p < 0.05, **p < 0.01, ***p < 0.001. Gender: 1 = male, 0 = female. SD, standard deviation.

### 3.3 Multiple mediating model analysis

**Figure 2:**
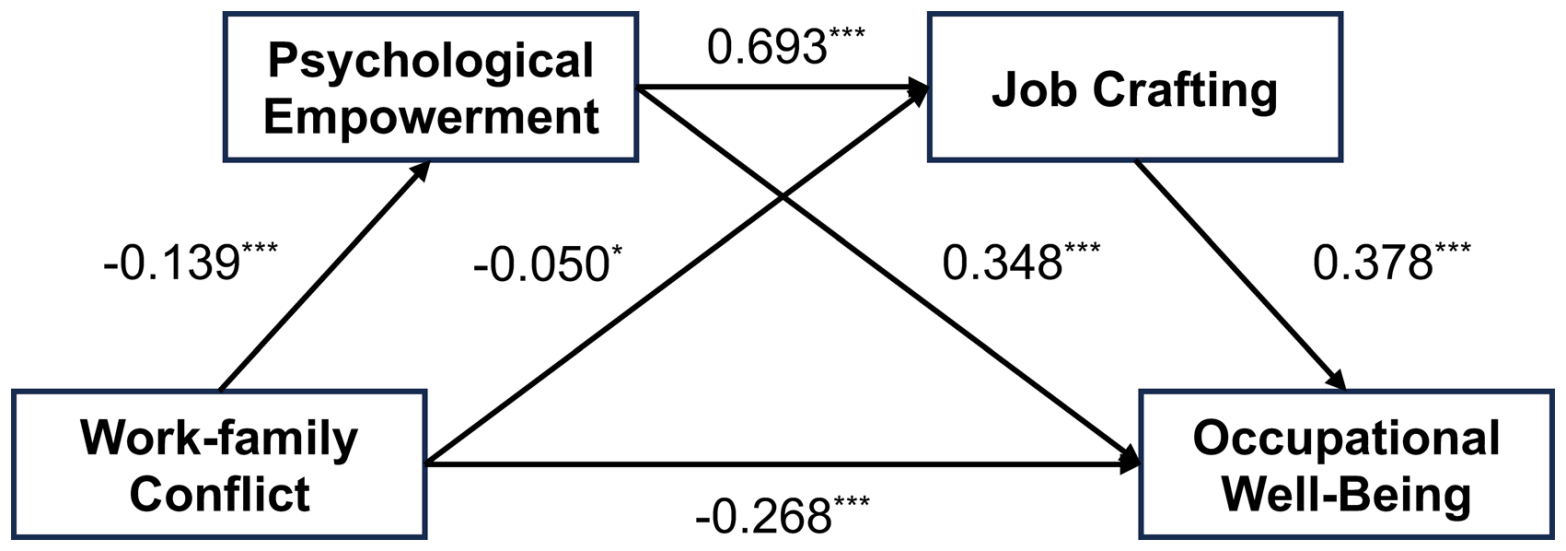
Chain mediation model testing the indirect links between work–family conflict and early childhood teachers’ occupational well-being via psychological empowerment and job crafting. *p < 0.05, ***p < 0.001. All coefficients are standardized.

After standardizing all variables, the SPSS macro PROCESS (Model 6) developed by Hayes was employed to test the mediating effects of psychological empowerment and job crafting on the relationship between work–family conflict and the occupational well-being of early childhood teachers. The regression equations included age, gender, and daily work hours as covariates. The results are presented in Table 2 and Figure 2.

**Table 2:** Regression analysis of work–family conflict and early childhood teachers’ occupational well-being.

Regression equation		Fitting index			Significance	
Result variable	Predictor variable	R	R2	*F*	*β*	*t*
Occupational well-being		0.435	0.189	69.378***		
	Gender				0.370	2.364*
	Age				0.018	5.500***
	Daily working hours				−0.085	−2.878**
	Work–family Conflict				−0.372	−13.988***
Psychological empowerment		0.186	0.034	10.612***		
	Gender				0.386	2.276*
	Age				0.010	2.721**
	Daily working hours				−0.040	−1.235
	Work–family conflict				−0.139	−4.824***
Job crafting		0.704	0.495	233.451***		
	Gender				0.182	1.473
	Age				0.001	0.224
	Daily working hours				−0.011	−0.455
	Work–family conflict				−0.050	−2.390*
	Psychological empowerment				0.693	32.950***
Occupational well-being		0.784	0.614	315.023***		
	Gender				0.066	0.611
	Age				0.012	5.250***
	Daily working hours				−0.057	−2.789**
	Work–family conflict				−0.268	−14.434***
	Psychological empowerment				0.348	13.606***
	Job crafting				0.378	14.827***

All variables used in the model have been standardized. The control variables include gender, age, and daily work hours. The independent variable is work–family conflict, and the outcome variable is early childhood teachers’ occupational well-being. *p < 0.05, **p < 0.01, ***p < 0.001.

The results of the linear regression analysis indicate that there is a significant correlation between work–family conflict and early childhood teachers’ occupational well-being (*β* = −0.268, *p* < 0.001), supporting Hypothesis 1. Work–family conflict significantly affects psychological empowerment (*β* = −0.139, *p* < 0.001) and job crafting (*β* = −0.050, *p* < 0.05). Psychological empowerment is significantly associated with job crafting (*β* = 0.693, *p* < 0.001) and occupational well-being (*β* = 0.348, *p* < 0.001). Additionally, job crafting is found to be a significant predictor of early childhood teachers’ occupational well-being (*β* = 0.378, *p* < 0.001).

Based on these findings, both psychological empowerment and job crafting play significant mediating roles in the relationship between work–family conflict and occupational well-being. Furthermore, this study also identified a significant chain mediation effect of psychological empowerment and job crafting between work–family conflict and occupational well-being, supporting Hypotheses 2 through 4.

To estimate the values of the mediating effects, 5,000 bootstrap samples were conducted at a 95% confidence level. The results showed that psychological empowerment and job crafting mediate the relationship between work–family conflict and early childhood teachers’ occupational well-being. As shown in Table 3, the 95% confidence intervals of the three indirect paths all exclude 0, indicating that: the indirect effect through psychological empowerment is significant (indirect effect size = −0.049); the indirect effect through job crafting is significant (indirect effect size = −0.019);the chain mediation effect of psychological empowerment and job crafting is significant (indirect effect size = −0.036); the total indirect effect of work–family conflict on occupational well-being was −0.104, accounting for 28.03% of the total effect. This again confirms Hypotheses 2 through 4.

**Table 3:** Analysis of the mediating effects of psychological empowerment and job crafting.

	Indirect effects	Boot SE	Boot LLCI	Boot ULCI	Relative mediation effect
Total indirect effect	−0.104	0.028	−0.160	−0.051	28.03%
Indirect effect 1	−0.049	0.015	−0.081	−0.019	13.21%
Indirect effect 2	−0.019	0.009	−0.038	−0.001	5.12%
Indirect effect 3	−0.036	0.012	−0.060	−0.014	9.70%

Indirect Effect 1: Work–Family Conflict → Psychological Empowerment → Occupational Well-being; Indirect Effect 2: Work–Family Conflict → Job Crafting → Occupational Well-being; Indirect Effect 3: Work–Family Conflict → Psychological Empowerment → Job Crafting → Occupational Well-being. LL, lower limit; CI, confidence interval; UL, upper limit; SE, standard error.

## 4 Discussion

In summary, this study established a chain mediation model based on the JD-R model and SDT to explore the correlation between work–family conflict and early childhood teachers’ occupational well-being. In addition to direct impacts, the effect of work–family conflict on occupational well-being may operate through three mediating pathways: (1) via the mediation of psychological empowerment, (2) via the mediation of job crafting, and (3) through a chain mediation of psychological empowerment and job crafting. Thus, psychological empowerment and job crafting partially mediate the relationship between work–family conflict and occupational well-being. This will help us to further understand the relationship between work–family conflict and the occupational well-being of early childhood teachers, as well as help us to appropriately adjust our plans to further improve the occupational well-being of early childhood teachers.

### 4.1 The relationship between work–family conflict and early childhood teachers’ occupational well-being

The results revealed a negative correlation between work–family conflict and the occupational well-being of early childhood teachers, consistent with previous research (24, 57, 87). Specifically, positive work-family relationships may significantly enhance the occupational well-being of early childhood teachers. This harmonious state is reflected in the support from families for work, the balance between work and life, and the mutual promotion between the two. For example, the empathy and emotional insight cultivated by early childhood teachers in their work enable them to more keenly perceive the emotional needs of their family members in family life, thereby providing timely emotional support and comfort, and fostering harmony and stability within the family. Concurrently, understanding, encouragement, and shared responsibilities from family members can effectively alleviate the work-related stress of teachers (24). In situations where work and family resources mutually enhance each other, early childhood teachers tend to exhibit higher job enthusiasm and greater work commitment, leading to a significant boost in their professional fulfillment and occupational well-being. However, negative work-family relationships can have a detrimental effect on the occupational well-being of early childhood teachers. This negative state manifests as conflicts between work and family, the accumulation of stress, and emotional alienation. Furthermore, according to the JD-R model, the higher the level of work–family conflict, the higher the level of job demands, and the relatively insufficient available resources (such as time, energy, support, etc.) (59). This imbalance can lead to psychological strain among early childhood teachers. Over time, this psychological strain can increasingly negatively impact the occupational well-being of early childhood teachers, such as reduced job satisfaction.

Currently, with increasing expectations and demands in education, early childhood teachers not only perform caregiving and educational roles but also face more frequent inspections and assessments. These often involve complex tasks like environmental setups and continuous documentation (49, 88–91). Such accumulating pressures greatly increase the likelihood of work–family conflict among early childhood teachers (92). According to the JD-R model and related studies, when individuals encounter work–family conflict, such conflict can lead to increased stress and fragmented time, making it difficult for individuals to find a balance between work and family, which leads to tension, anxiety, fatigue, and dissatisfaction. This, in turn, triggers health issues and lowers occupational well-being (57, 93, 94).

Secondly, Chinese early childhood teachers may experience higher work–family conflict due to traditional cultural influences. Specifically, China has always emphasized home culture, wherein the family is regarded as a crucial component of individual life and a primary source of emotional belonging and social support (95). This results in early childhood teachers not only needing to play multiple roles at work, such as educators, caregivers, observers, and environment creators, but also requiring them to fulfill key roles within the family as parents, children, and other significant figures, striving to maintain family harmony and happiness. When early childhood teachers lack adequate resources or face difficulties in restructuring their work, this dual-role stress exacerbates their vulnerability to work–family conflict, resulting in fatigue, decreased job satisfaction, and ultimately, a negative impact on their occupational well-being. Meanwhile, due to the influence of traditional cultural ideologies, Chinese individuals tend to view themselves more as collective members within social networks and attach greater importance to harmonious interpersonal relationships within these networks. Emphasizing harmony and tolerance makes teachers less willing to confront management directly to reduce excessive workloads and the interpersonal stress. However, this pursuit of harmony may lead to more unresolved stress and exhaustion, resulting in greater fatigue and burnout, threatening their occupational well-being (24, 96). This aligns with the JD-R model’s “health impairment process” (57).

### 4.2 The mediating role of psychological empowerment in the relationship between work–family conflict and early childhood teachers’ occupational well-being

The findings of this study indicate that psychological empowerment mediates the impact of work–family conflict on early childhood teachers’ occupational well-being, aligning with the theoretical hypothesis. When early childhood teachers possess higher levels of psychological empowerment, they may exhibit greater psychological resilience and coping abilities, leading them to adopt more confident and proactive measures to alleviate work–family conflict, such as rational workload allocation and effective communication with kindergarten leaders and family members. This approach helps to mitigate the stress and tension arising from work–family conflict, thereby reducing its adverse effects on the occupational well-being of early childhood teachers (45). However, in situations where the level of psychological empowerment is limited or low, and work–family conflict is high, the accompanying stress can further diminish psychological empowerment, subsequently jeopardizing the occupational well-being of early childhood teachers. Specifically, when early childhood teachers struggle with work–family conflict and experience the cumulative stress of their profession, their sense of self-efficacy may be compromised, rendering them less capable of effectively dealing with the dual challenges of work and life. Secondly, China’s educational system emphasizes centralized management and accountability (97). Within this context, early childhood teachers often experience heightened levels of control. When confronted with work–family conflict, this sense of being controlled by both work and life may intensify. Concurrently, in the context of conflict, early childhood teachers’ perception of the meaning of their work may become blurred, and their influence and sense of achievement in the work environment may significantly decrease. These manifestations of low levels of psychological empowerment collectively form a negative feedback loop, further eroding the occupational well-being of early childhood teachers (46, 47). JD-R model also provides another analytical perspective on this impact mechanism. When early childhood teachers confront work–family conflict amidst limited job resources, the high stress resulting from this conflict may lead to excessive depletion of their energy and decreased motivation. Consequently, early childhood teachers’ psychological empowerment, as a motivational construct, is adversely affected (47). The findings of this study further validate the JD-R model, revealing the underlying mechanism through which work–family conflict influences early childhood teachers’ occupational well-being via psychological empowerment.

Moreover, this study is the first to systematically examine the mediating role of psychological empowerment among early childhood teachers in the relationship between work–family conflict and occupational well-being, filling a gap in this research area. Prior studies have largely focused on the impact of single variables or direct relationships, whereas this study introduces psychological empowerment as a mediating variable, offering a new perspective and evidence for understanding the complex antecedents and consequences of early childhood teachers’ occupational well-being.

### 4.3 The mediating role of job crafting in the relationship between work–family conflict and early childhood teachers’ occupational well-being

The findings of this study indicate that job crafting mediates the impact of work–family conflict on the occupational well-being of early childhood teachers, which aligns with our theoretical hypothesis. Specifically, the profession of early childhood teachers necessitates a high level of patience, responsibility, and empathy to meet the diverse needs and contingencies of young children. The particular nature of this work requires teachers to devote substantial time and energy. When faced with work–family conflict, they struggle to have the adequate time and energy to engage in job crafting. According to the JD-R model, high workloads may make it challenging for early childhood teachers to find sufficient energy to cope with additional demands, prompting them to avoid job crafting—that is, reducing proactive behaviors aimed at adjusting and optimizing their work environment and tasks (57).

Meanwhile, from the perspective of Conservation of Resources (COR) theory, when facing resource depletion (such as time and energy squeezed by dual pressures from family and work), early childhood teachers may adopt a series of strategies to minimize further losses. These include altering their work attitudes, such as reducing enthusiasm and commitment to their work, and decreasing positive behaviors, like attempts at job crafting. These adjustments may seem to help alleviate stress but, in reality, can lead to a significant decline in occupational well-being (98). The findings of this study further validate the aforementioned models and theories and successfully reveal the mediating role of job crafting between work–family conflict and occupational well-being, addressing a gap in previous research.

### 4.4 The chain mediating effect of psychological empowerment and job crafting on the relationship between work–family conflict and early childhood teachers’ occupational well-being

Results also showed that psychological empowerment and job crafting have a chain mediating effect on the relationship between work–family conflict and early childhood teachers’ occupational well-being, aligning with theoretical assumptions. This process can be interpreted considering the actual situation faced by early childhood teachers. Their energy is limited, and excessive work pressures force many to divert attention from education to handle non-teaching tasks like environmental setups and inspections (88). These complex non-teaching tasks not only affect their teaching time and organization of outdoor activities but also intrude on family care, such as time with family, children’s education, and household chores, leading to work–family conflict. Without proper support and coping mechanisms, such conflicts increase feelings of helplessness, reduce proactivity, and hinder occupational well-being (58, 99). Compared to previous studies, this study incorporated psychological empowerment and job crafting to explore the mechanism by which work–family conflict affects early childhood teachers’ occupational well-being, addressing a gap in existing research. Additionally, the positive influence of psychological empowerment and job crafting provides a strong foundation for future actions aimed at enhancing early childhood teachers’ occupational well-being.

### 4.5 Theoretical and practical implications

Despite the limitations, our results have theoretical and practical significance. Theoretically, this study focused on how work–family conflict impacts occupational well-being among early childhood teachers. Combining the JD-R and SDT, this study constructed the “work–family conflict→psychological empowerment→job crafting→occupational well-being” model to elucidate the effects of work–family conflict on the occupational well-being of early childhood teachers. Second, this study further validates the JD-R model and adds new findings. Research has shown that the dual processes of the JD-R model are not completely independent (56). This study constructs the model in conjunction with the dual processes of the JD-R model, and then explores the relationships between the different variables, which is a further exploration of the mechanism of action between the variables of this theoretical model. Furthermore, this study offers new evidence about the relationship between work–family conflict and occupational well-being in early childhood teachers under the Chinese cultural context. It is worth mentioning that previous research mainly focused on employee, psychological, and subjective well-being but rarely on early childhood teachers’ occupational well-being. Few studies examined work–family conflict’s influence on well-being, or investigated psychological empowerment and job crafting together. Thus, this study fills this research gap, providing theoretical and practical foundations. Lastly, it discussed inconsistencies in research findings regarding the influence of work–family conflict to job crafting, encouraging further exploration.

Practically, the study is valuable in developing strategies to improve early childhood teachers’ occupational well-being. As work–family conflict negatively predicts occupational well-being, educational administrators, school managers, and teachers’ families should recognize potential work–family conflict and take measures to reduce stress. At the management level, kindergarten administrators should first implement flexible work arrangements, including elastic working hours that permit teachers to adjust their schedules or work remotely in specific circumstances (e.g., family emergencies, child illness) (30), to accommodate personal work-life balance needs. Where feasible, staggered working hours could also be piloted. Secondly, a temporary substitute teacher program should be established, comprising a pool of substitute teachers or assistants, to relieve duties when teachers need to attend to family matters. Additionally, administrators should optimize workload management by regularly assessing teachers’ workload and stress levels through surveys or interviews, and subsequently tailor optimizations based on individual circumstances. At the family level, enhanced communication and understanding among family members are crucial. Regular family meetings can be held to share recent routines and challenges, and collectively discuss solutions (100). Secondly, teachers and their families can regularly plan family activities, such as weekend outings, parent–child games, or family movie nights, to strengthen emotional bonds and provide teachers with opportunities to relax and recharge. Meanwhile, family members should offer appropriate emotional support, patiently listening when teachers encounter work stress or family conflicts, encouraging them to share feelings, and providing positive feedback and advice (101, 102). Moreover, the chain mediation model offers another way to enhance occupational well-being by increasing psychological empowerment and encouraging job crafting to improve resources. Therefore, kindergartens should prioritize the psychological empowerment of these teachers by providing training, mentorship, and support to enhance their sense of control and self-efficacy in their work. Additionally, kindergartens should offer flexible work arrangements, enabling early childhood teachers to adjust their working hours and tasks according to their personal circumstances, thereby facilitating a better balance between work and family demands.

### 4.6 Limitations and future research directions

The study has some limitations. First, using convenience sampling, data were collected from over a thousand teachers in Guangdong Province but did not include participants from other regions. This sampling approach may introduce potential selection bias (103), leading to discrepancies in demographic characteristics, cultural backgrounds, and teaching experiences between the sample and the broader population of early childhood teachers. For instance, teachers in economically developed regions may have access to more educational resources and professional development opportunities, whereas those in less developed areas may face greater teaching challenges and resource constraints. These differences could limit the generalizability of our findings. Consequently, our results may not fully represent all early childhood teachers, particularly those from different provinces or countries. Future research should consider adopting broader sampling methods, such as random or stratified sampling, to ensure sample representativeness. Additionally, cross-cultural and cross-national studies are effective means of enhancing generalizability, allowing for a more comprehensive understanding of the commonalities and diversities in early childhood teachers’ experiences across different cultural, economic, and policy contexts.

Second, the cross-sectional design does not establish direct causality. However, bidirectional or reverse causal relationships often exist between some variables, for instance, when changes occur in psychological empowerment and job crafting, the occupational well-being of early childhood teachers may potentially experience improvement. Such enhancement may, in turn, alleviate the pressures stemming from familial roles, thereby reducing work–family conflict. To clarify causal relationships between variables, future research could adopt a longitudinal design, collecting data on variables at different time points and applying potential growth model or cross-lag model.

Moreover, this study only selected work–family conflict as an independent variable and psychological empowerment and job crafting as mediating variables to investigate their mechanisms affecting occupational well-being. However, the influence of work-related factors is a complex process affected by various factors. Future research can further explore the influence of additional factors, such as emotional intelligence and organizational support, which serve as potential moderating variables, on this particular relationship, thereby enhancing our comprehensive understanding of the various facets of this complex mechanism. It is worth mentioning that when traditional familial roles undergo shifts (such as husbands taking on household responsibilities and older adult caring for children), the burdens on early childhood teachers within the family environment may potentially be eased, thereby granting them psychological empowerment to better cope with their workload. Future research may also delve into the influence of changes in familial roles on work–family conflict and occupational well-being among early childhood teachers.

Lastly, research varies on the influence of work–family conflict on job crafting. Some studies indicate a negative impact, while others find “positive aspects” (58, 104). The differing results may relate to the degree of work–family conflict. Moderate conflict may encourage proactive action, but excessive demands can overwhelm and negatively impact work attitudes and behavior (57). Future studies could explore the tipping point between positive and negative influences.

## 5 Conclusion

This study, considering the cultural context of China, thoroughly examined the impact of work–family conflict on early childhood teachers’ occupational well-being and its underlying mechanisms. It utilized the JD-R model and integrated SDT to construct a mediation model. Findings demonstrated that work–family conflict has a negative predictive effect on occupational well-being, and psychological empowerment and job crafting partially mediate this relationship. This study provides evidence supporting the significant impact of work-related stressors on occupational well-being and offers new insights for enhancing early childhood teachers’ well-being.

## Data Availability

The authors will provide unrestricted access to the raw data supporting the conclusions of this article.
